# Mechanical Properties of Dental Occlusal Splint Materials After Accelerated Aging in Artificial Saliva: An In Vitro Study

**DOI:** 10.3390/dj14080478

**Published:** 2026-08-04

**Authors:** Iulia Karla Nică, Lucian Toma Ciocan, Vlad Gabriel Vasilescu, Robert Cătălin Ciocoiu, Federico Foschi, Andreea Mihaela Custură, Elisei Adelin Radu, Alexandru Titus Farcașiu, Silviu Mirel Pițuru, Marina Imre

**Affiliations:** 1 Department of Prosthetics, Faculty of Dentistry, “Carol Davila” University of Medicine and Pharmacy, 020021 Bucharest, Romania; iulia.nica@umfcd.ro (I.K.N.); alexandru.farcasiu@umfcd.ro (A.T.F.); marina.imre@umfcd.ro (M.I.); 2 Discipline of Dental Prosthetics Technology, Faculty of Dentistry, “Carol Davila” University of Medicine and Pharmacy, Dionisie Lupu Street, No. 37, District 2, 020021 Bucharest, Romania; lucian.ciocan@umfcd.ro (L.T.C.); andreea-mihaela.custura@drd.umfcd.ro (A.M.C.); elisei-adelin.radu@drd.umfcd.ro (E.A.R.); 3 Department of Metallic Materials Science, Physical Metallurgy, University Politehnica of Bucharest, 313 Splaiul Independentei, J Building, 060042 Bucharest, Romania; 4 Eastman Dental Institute, Rockefeller Building, 21 University Street, London WC1E 6DGL, UK; f.foschi@ucl.ac.ukf; 5 Discipline of Organization, Professional Legislation and Dental Office Management, “Carol Davila” University of Medicine and Pharmacy, Dionisie Lupu Street, No. 37, District 2, 020021 Bucharest, Romania; silviu.pituru@umfcd.ro

**Keywords:** occlusal splint, accelerated aging, SLA 3D printing, photopolymer resin, Shore D hardness, flexural properties, PMMA, PETG, three-point bending, artificial saliva

## Abstract

**Background**: Occlusal splints are exposed to prolonged intraoral conditions, including saliva, temperature fluctuations, and mechanical loading, which may alter their mechanical performance over time. The increasing use of SLA 3D-printed photopolymer resins for splint fabrication has introduced new materials whose aging-related mechanical stability remains insufficiently characterized. **Objectives**: This in vitro study evaluated the effects of accelerated aging in artificial saliva on the Shore D hardness and flexural properties, including flexural modulus, flexural strength, and strain at failure, of four polymeric materials used for occlusal splint fabrication: a thermoformed PETG material (Duran, C), a milled PMMA (Bilkim Polywax, F), and two SLA-printed photopolymer resins (HARZ Labs Dental Clear, H; NextDent Ortho Rigid, N). **Materials and Methods**: Eighty rectangular specimens (*n* = 20/material) were fabricated following ASTM D790 and subjected to accelerated aging in Fusayama artificial saliva at 60 °C for 24, 48, and 72 h. Shore D hardness was assessed first as a non-destructive measurement, followed by three-point bending tests yielding flexural modulus, flexural strength, and strain at failure. Data were analyzed using one-way ANOVA and post hoc multiple comparisons at a significance level of α = 0.05. **Results**: Aging effects were material-dependent. Duran (C) showed no statistically significant changes in Shore D hardness (*p* = 0.651) and only non-progressive variations in flexural behavior. Bilkim Polywax (F) exhibited a significant hardness reduction at 48 h (*p* = 0.004) and selective changes in flexural strength at 72 h. HARZ Labs Dental Clear (H) showed the most severe and progressive deterioration, with significant reductions in all evaluated parameters, including hardness decreases of up to 5.8% and flexural modulus reductions exceeding 49%. NextDent Ortho Rigid (N) demonstrated an initial hardness decrease followed by stabilization, with a significant reduction in flexural modulus at 72 h. **Conclusions**: Under the accelerated hydrothermal aging conditions employed in this study, the two investigated SLA-printed photopolymer resins (HARZ Labs Dental Clear and NextDent Ortho Rigid) exhibited greater mechanical deterioration than the tested thermoformed PETG and milled PMMA materials. These findings are limited to the investigated materials, standardized rectangular specimens, and the specific accelerated aging protocol used in this study. Among the tested materials, thermoformed PETG demonstrated the greatest mechanical stability. Material-specific evaluation of aging resistance remains advisable before routine clinical application, particularly for digitally fabricated occlusal splints intended for prolonged intraoral service.

## 1. Introduction

Occlusal splints are among the most widely used removable dental appliances in clinical practice, with indications including temporomandibular disorders, bruxism, and sleep-related parafunctional activity, as well as applications in prosthodontic and orthodontic retention. Recently, there has been mounting evidence supporting their utilization following endodontic treatment to minimize the incidence of catastrophic fractures [1]. Their clinical effectiveness depends on maintaining dimensional accuracy, surface integrity, and mechanical stability throughout the intended period of use, which may range from several months to multiple years [2]. Prolonged intraoral exposure subjects splint materials to mechanical loading, thermal cycling, and chemical interactions with salivary enzymes and buffering components, all of which may contribute, either collectively or independently, to material degradation over time [3,4].

Traditionally, occlusal splints have been fabricated using thermoforming of polymeric sheets over dental casts, and polymerization of acrylic resins, either through conventional heat- or cold-curing methods. Among the materials most widely employed are polyethylene terephthalate glycol (PETG), a thermoplastic with favorable optical properties and processing characteristics, and polymethylmethacrylate (PMMA), which offers established mechanical performance and clinical predictability [5,6]. In recent years, milled PMMA disks produced using computer-aided design and computer-aided manufacturing (CAD/CAM) workflows have gained widespread adoption, offering improved material homogeneity and reproducibility compared to conventionally processed acrylic [7].

The integration of additive manufacturing into dental workflows has introduced an additional category of materials for splint fabrication: photopolymer resins designed for use with digital light processing (DLP) or stereolithography (SLA) 3D printing systems. These materials offer potential advantages in terms of workflow efficiency, geometric flexibility, and customization; however, concerns remain regarding their long-term stability under intraoral conditions [8,9]. Compared with conventional thermoformed or milled materials, SLA-printed photopolymers may exhibit higher water sorption, residual monomer content, layer-dependent anisotropy, and variability related to printing orientation and post-curing procedures. These factors may substantially influence their mechanical durability and aging behavior, particularly during prolonged clinical service [10,11].

Evaluating the mechanical stability of dental materials under realistic clinical conditions poses significant methodological challenges, particularly when considering extended time frames. Accelerated aging protocols, as described in ASTM F1980-07 and referenced in ISO 11607-1:2019, provide a validated framework for simulating the time-dependent effects of environmental exposure in a controlled and reproducible manner [12,13]. Immersion in artificial saliva at elevated temperature is widely employed as a controlled hydrothermal aging model, as it reproduces water sorption, ionic interactions, and polymer plasticization processes that may contribute to time-dependent mechanical degradation of dental polymers. The Fusayama artificial saliva formulation has been extensively used in dental materials research due to its compositional similarity to human whole saliva [14,15,16].

Shore D hardness and three-point bending tests, conducted in accordance with ASTM D2240 and ASTM D790, respectively, represent well-established methodologies for the mechanical characterization of polymeric dental materials [17,18]. Shore D hardness provides a rapid, non-destructive measure of surface resistance that correlates with material stiffness and wear resistance, while the three-point bending configuration enables the determination of flexural modulus, flexural strength, and strain at failure under standardized loading conditions [17]. These parameters are clinically relevant in the context of occlusal splint performance, as they reflect the capacity of the material to resist deformation under functional loading and to maintain structural integrity during prolonged use [18,19].

Despite the growing clinical use of digitally manufactured occlusal splints, direct comparison information for the aging-related mechanical stability of thermoformed, milled, and SLA-printed materials under standardized settings is limited. Few studies have specifically examined whether general-purpose SLA photopolymers and specialized splint resins show similar resistance to mechanical deterioration and hydrothermal degradation following extended exposure to simulated intraoral environments. This restriction makes choosing materials for long-term clinical applications more difficult and emphasizes the necessity of standardized comparative studies. The present study addresses this gap by evaluating four representative materials spanning the principal fabrication categories currently in clinical use. The aim of this in vitro study was to assess the effects of accelerated aging in Fusayama artificial saliva at 60 °C on the Shore D hardness and flexural properties (flexural modulus, flexural strength, and strain at failure) of a thermoformed PETG material (Duran), a milled PMMA material (Bilkim Polywax), and two SLA-printable dental resins intended for the fabrication of occlusal splints and dental appliances (NextDent Ortho Rigid and HARZ Labs Dental Clear), at 24, 48, and 72 h of exposure. Although both NextDent Ortho Rigid and HARZ Labs Dental Clear are marketed for the fabrication of dental appliances, including splints and night guards, they differ in formulation, manufacturer-reported mechanical properties, and clinical positioning. Therefore, both materials were included to evaluate whether distinct SLA-printable photopolymer systems exhibit different aging-related mechanical responses under identical hydrothermal conditions.

The null hypothesis was that no statistically significant differences would be observed in mechanical properties between baseline and post-aging measurements across the tested materials and time points.

## 2. Materials and Methods

### 2.1. Materials

Four polymeric materials representing distinct fabrication categories were selected for evaluation. Duran (C) (Scheu Dental GmbH, Iserlohn, Germany) is a transparent PETG-based thermoplastic sheet material indicated for thermoforming over dental casts. It is characterized by low water absorption, high biocompatibility, and resistance to fracture. Bilkim Polywax (F) (Bilkim Dental Products, Ankara, Türkiye) is a milled PMMA-based material presenting a manufacturer-reported flexural strength of approximately 105.91 MPa and water absorption of 13.40 µg/mm^3^, compliant with ISO 10477. HARZ Labs Dental Clear (H) (HARZ Labs LLC, Moscow, Russia) is a transparent SLA-printable photopolymer resin with a reported flexural strength of 120.0 ± 20.0 MPa and flexural modulus of 2000 ± 156 MPa per ASTM D790, indicated for the fabrication of splints, night guards, and other dental appliances. NextDent Ortho Rigid (N) (Vertex-Dental B.V., Soesterberg, The Netherlands) is a biocompatible Class IIa SLA-printable resin specifically developed for the digital fabrication of occlusal splints and orthodontic appliances, with a reported flexural strength of ≥78 MPa and flexural modulus of 2075 MPa per ISO 20795-2 [19]. Material composition and manufacturer-specified properties are summarized in Table 1.

### 2.2. Specimen Fabrication

A total of 80 rectangular specimens were fabricated (*n* = 20 per material) with mean dimensions of 12 mm in width, 3 mm in thickness, and 80 mm in length, conforming to the requirements of ASTM D790 for three-point bending evaluation. Each set of 20 specimens per material was divided into four subgroups of 5 specimens, corresponding to the four aging conditions (reference, 24 h, 48 h, and 72 h). Shore D hardness measurements were performed on all specimens within each subgroup prior to the three-point bending test, exploiting the non-destructive nature of durometer evaluation to allow sequential mechanical characterization of the same specimens.

Duran^®^ PETG specimens were fabricated from transparent thermoforming sheets (1.5 × 125 mm; lot no. 0424A; Scheu-Dental GmbH, Iserlohn, Germany). Thermoforming was performed using a Model 202 vacuum-forming machine (T&S Dental & Plastics, Keystone Industries, Myerstown, PA, USA). The thermoforming cycle followed the manufacturer’s recommendations, consisting of approximately 40 s of heating followed by 120 s of cooling.

Bilkim Polywax PMMA (F) specimens were fabricated from pre-polymerized monolayer PMMA disks (98 × 18 mm, shade A1, lot no. BLH-241244321-25). Specimens were machined using an imes-icore 250i CAD/CAM milling system (imes-icore GmbH, Eiterfeld, Hesse, Germany) operating in simultaneous 5-axis mode. Milling was performed using dedicated PMMA burs of 2 mm and 1 mm diameter for rough and finishing machining, respectively. Following milling, the specimens were separated from the CAD/CAM disk, and residual connectors (sprues) were carefully removed. The specimens were subsequently rinsed with distilled water and allowed to dry at room temperature prior to mechanical testing. No additional polishing procedures intended to modify the surface characteristics of the material were performed prior to testing.

The SLA-printed specimens (HARZ Labs Dental Clear and NextDent Ortho Rigid) were fabricated using an xPRINT 8K Ultra HD LCD 3D printer (xDEPOT GmbH, Dachau, Germany). Specimens were printed with a build orientation of 45° relative to the build platform, with the occlusal surface oriented toward the build plate. A layer thickness of 100 μm was selected according to the manufacturer’s recommendations. The printing parameters included an exposure time of 4.7 s per layer, a retraction distance of 8 mm, a retraction speed of 180 mm/min, and a lifting speed of 120 mm/min. Following printing, the specimens were rinsed with 99% isopropyl alcohol and subsequently washed in fresh 99% isopropyl alcohol for 6 min. Post-curing was performed using a Formlabs Form Cure L unit (70 W LED power, Formlabs Inc., Somerville, MA, USA). The specimens were first cured at 70 °C, followed by a second thermal stage consisting of 30 min at 80 °C. After post-processing, the specimens were stored in opaque, hermetically sealed containers at room temperature until mechanical testing.

All specimens were inspected under magnification for surface defects prior to inclusion and measured with a digital caliper to confirm dimensional compliance.

### 2.3. Accelerated Aging Protocol

Accelerated aging was performed by immersion in Fusayama-Meyer artificial saliva as an electrolyte, with the following chemical composition: 0.4 gL^−1^ NaCl, 0.9 gL^−1^ KCl, 1 gL^−1^ urea, 0.69 gL^−1^ NaH_2_PO_4_, 0.795 gL^−1^ CaCl**·**2H_2_O (pH 5.2) at a constant temperature of 60 °C using a thermostatically controlled water bath, in accordance with ASTM F1980-07 (2011). All immersed specimens were introduced at the same time in the same thermostatic bath, five of each material being removed after every 24 h. All specimens were immersed collectively in 1 L of Fusayama artificial saliva, resulting in an approximate solution volume of 16.7 mL per specimen. Instead of directly simulating clinical time, the accelerated aging protocol used in this study was designed as a controlled hydrothermal stress model. In polymer-based dental materials, elevated-temperature immersion methods are frequently employed to speed up water diffusion, polymer plasticization, and hydrolytic breakdown processes. There is no widely recognized acceleration factor or activation energy for SLA-printable dental photopolymers exposed to artificial saliva, even though ASTM F1980-07 offers a broad framework for accelerated aging techniques. Therefore, the current methodology should be seen as a standardized comparison tool for assessing relative material susceptibility to hydrothermal deterioration under enhanced laboratory circumstances rather than as a precise equivalent of long-term intraoral aging.

Each material set of 20 specimens was divided into four subgroups of five specimens each, corresponding to the following experimental conditions: reference (untreated control), 24 h immersion, 48 h immersion, and 72 h immersion. The 24, 48, and 72 h intervals were chosen to provide sequential early, intermediate, and extended exposure conditions, allowing the assessment of time-dependent mechanical changes among the tested materials. Since there is no widely recognized activation energy or Q10 value for the investigated dental polymers under immersion in Fusayama artificial saliva, neither an acceleration factor nor a real-time clinical equivalency was computed. The immersion protocol was conducted without interruption at the assigned time points. Following aging, specimens were removed from the bath, gently blotted dry, and allowed to equilibrate at room temperature for 30 min before mechanical testing. The specimen coding employed the material identifier followed by the aging condition: R (reference), 24, 48, and 72, yielding designations such as CR, C24, C48, and C72 for the Duran material.

### 2.4. Mechanical Testing

Shore D hardness was assessed using an analog Shore D durometer operated manually with a resolution of 0.5 units, in accordance with ASTM D2240. Given the non-destructive nature of the measurement, ten independent determinations were performed on each specimen within the subgroup (*n* = 5 per condition per material; two readings per specimen), yielding a total of *n* = 10 values per condition.

Flexural strength (σ) and flexural modulus (*E*) were calculated using standard equations:

(1) $$\sigma =\frac{3FL}{2bd^{2}}$$

(2) $$E=\frac{FL^{3}}{4bd^{3m}}$$

where *F* is the applied load, *L* is the support span, *b* is the specimen width, d is the thickness, and m is the slope of the linear region of the load–deflection curve.

This approach allowed sequential evaluation of hardness and flexural properties on the same specimens, thereby eliminating inter-specimen variability between the two test phases. Three-point bending tests were subsequently performed on the same specimens using an Instron 34TM10 universal testing machine(Instron, Norwood, MA, USA) equipped with a three-point bending fixture with a support span of 50 mm, corresponding to a span-to-depth ratio of approximately 16:1. The selected support span resulted in a span-to-depth ratio of approximately 16:1, consistent with ASTM D790 recommendations for flexural testing of polymeric materials, minimizing shear effects and ensuring bending-dominated failure behavior. The support and loading nose radii were both 5 ± 0.1 mm. The crosshead speed was set at 1 mm/min, and all tests were conducted at a temperature of 24 ± 2 °C. Flexural strain at failure (εf) was calculated using the ASTM D790 relationship:

(3) $$\varepsilon f=\frac{6Dd}{L^{2}}$$

where D is the maximum mid-span deflection (mm), d is the specimen thickness (mm), and L is the support span (mm).

Five specimens per subgroup were tested (*n* = 5 per condition per material). From the resulting load–displacement curves, the flexural modulus, flexural strength, and strain at failure were calculated according to the standard equations. For material F (Bilkim Polywax PMMA), which exhibited ductile behavior without complete fracture, strain at failure was recorded at a predefined deformation threshold; the parameter is reported for completeness but acknowledged to have limited clinical significance given the non-catastrophic failure mode.

### 2.5. Statistical Analysis

All measurements were recorded and processed using dedicated statistical software. Descriptive statistics, including mean, standard deviation, median, minimum, and maximum values, were calculated for each subgroup. Normality was assessed prior to inferential testing. Within-material comparisons across aging conditions were performed using one-way analysis of variance (ANOVA). Where statistically significant differences were identified (α = 0.05), post hoc multiple comparisons were conducted using Tukey, Scheffé, Bonferroni, Holm, and Sidak tests to provide comprehensive coverage across different correction assumptions. The significance level was set at α = 0.05 for all analyses.

## 3. Results

### 3.1. Shore D Hardness

Shore D hardness results are presented separately for each material across the four aging conditions.

Material C (Duran—PETG):

Mean values ranged from 96.80 (C72) to 97.25 (C48) (Table 2), with narrow standard deviations throughout all conditions. The one-way ANOVA indicated no statistically significant differences among the four subgroups (F = 0.551; *p =* 0.651), confirming that accelerated aging did not significantly alter the Shore D hardness of this material within the evaluated time range (Table 3).

Material F (Bilkim Polywax—PMMA):

Mean Shore D hardness decreased from 89.25 at baseline to 88.20 after 24 h and reached a minimum of 87.40 after 48 h, followed by a partial recovery at 72 h (88.20) (Table 4). One-way ANOVA (Table 5) demonstrated statistically significant differences among aging conditions (F = 5.283, *p* = 0.004). Post hoc analysis identified significant differences only between baseline and F48.

Material H (HARZ Labs Dental Clear—SLA resin):

Material H exhibited a progressive reduction in Shore D hardness throughout the aging protocol, decreasing from 95.10 at baseline to 89.60 after 72 h (−5.8%) (Table 6). One-way ANOVA (Table 7) showed highly significant differences among ageing conditions (F = 95.22, *p* < 0.001), while post hoc analysis confirmed significant differences between all pairwise comparisons.

Material N (NextDent Ortho Rigid—SLA resin):

Material N showed an initial reduction in Shore D hardness after 24 h, followed by stabilization at 48 and 72 h (Table 8). One-way ANOVA (Table 9) identified significant overall differences (F = 12.00, *p <* 0.001). Post hoc analysis showed that baseline differed significantly from all aged groups, whereas no significant differences were observed among the aged subgroups.

**Figure 1:**
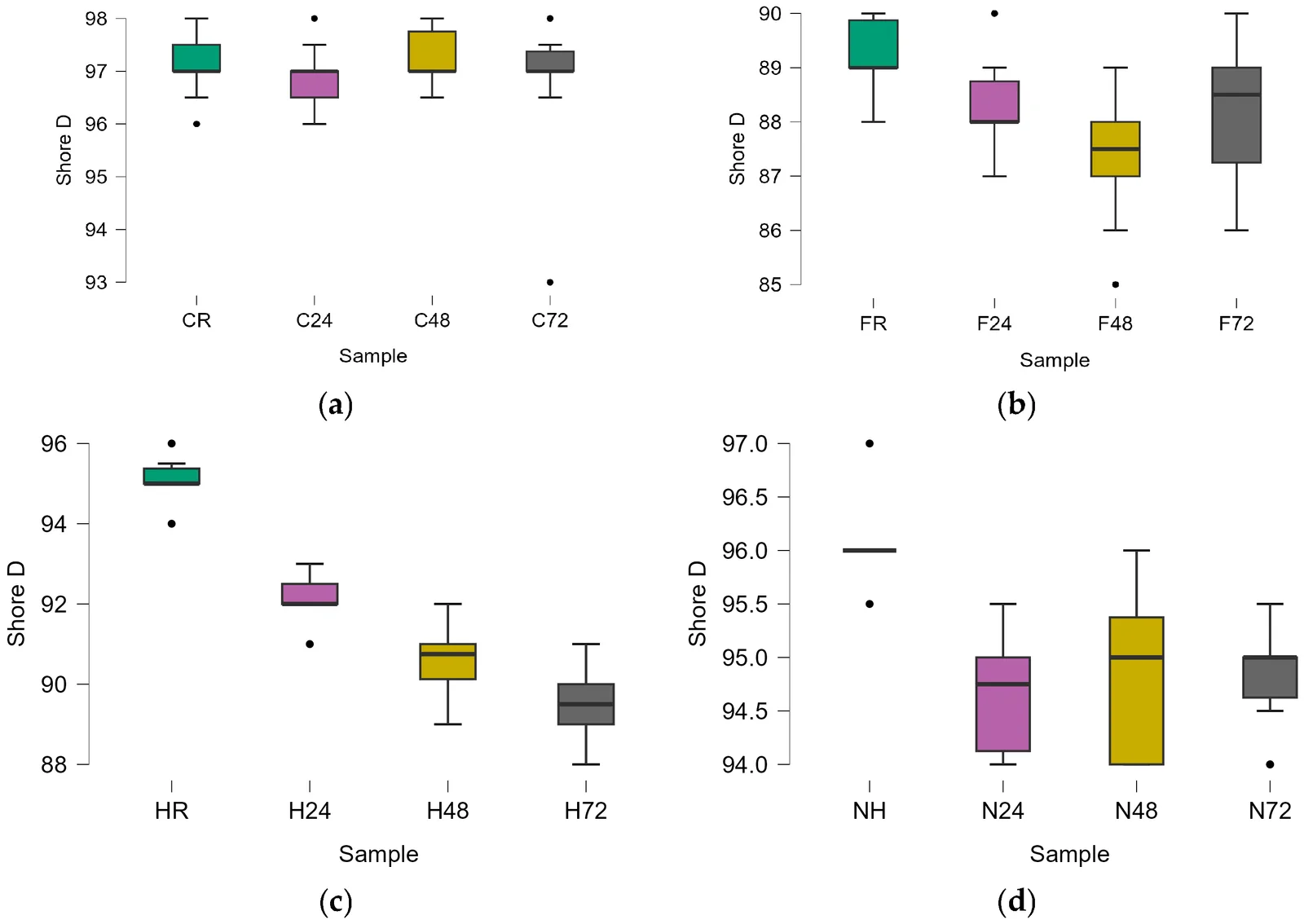
Box plot representation of Shore D hardness values for each material across aging conditions: (**a**) Duran (C), (**b**) Bilkim Polywax (F), (**c**) HARZ Labs Dental Clear (H), and (**d**) NextDent Ortho Rigid (N). The central line represents the median, boxes indicate interquartile range (IQR), whiskers represent minimum and maximum values, and individual points indicate outliers.

Box plot representation of Shore D hardness values (Figure 1) for each material across aging conditions: (a) Duran (C), (b) Bilkim Polywax (F), (c) HARZ Labs Dental Clear (H), and (d) NextDent Ortho Rigid (N). The central line represents the median, boxes indicate interquartile range (IQR), whiskers represent minimum and maximum values, and individual points indicate outliers.

**Figure 2:**
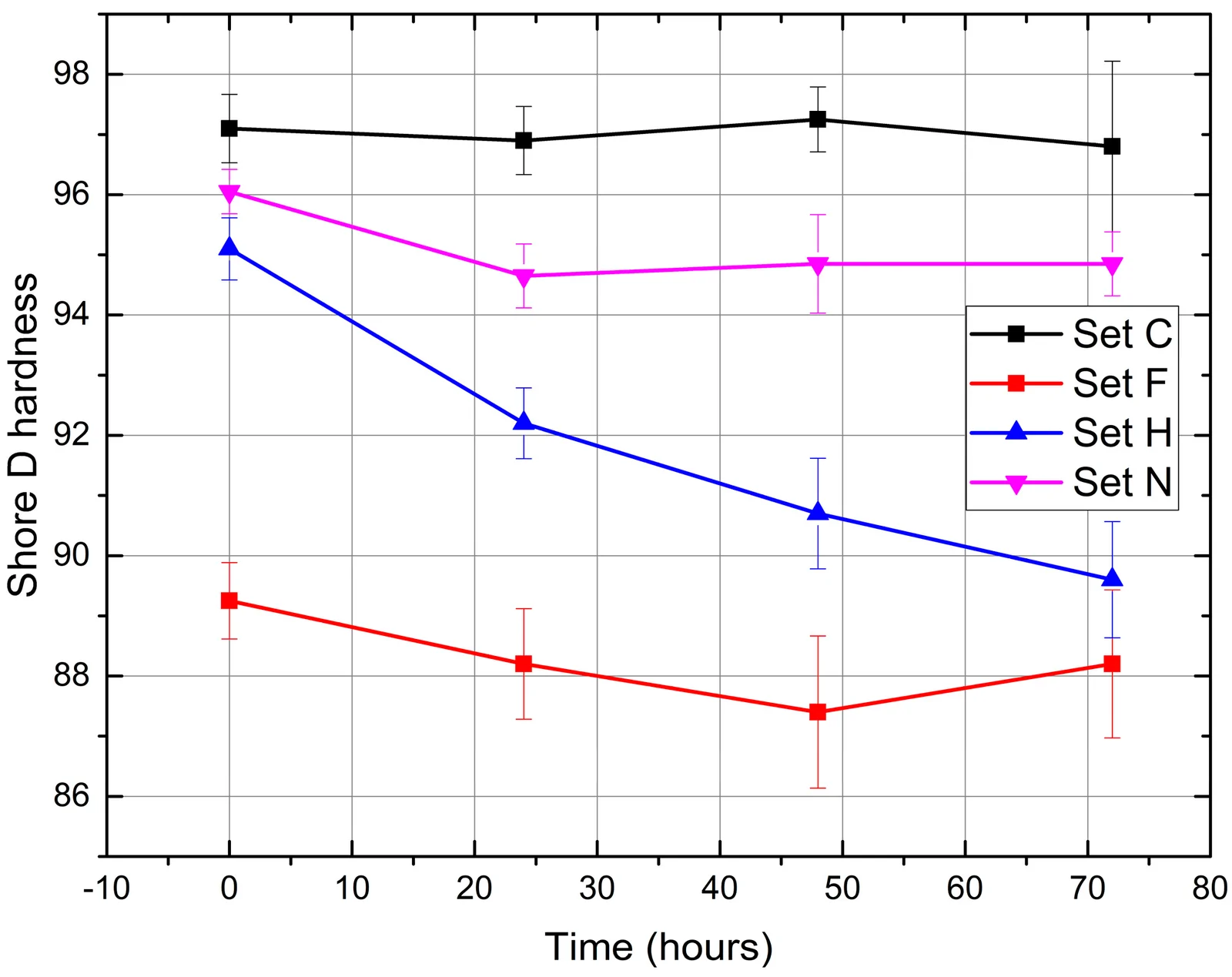
Variation in Shore D hardness values for the tested materials (C, F, H, N) as a function of accelerated aging time (CR, 24 h, 48 h, 72 h).

Figure 2 summarizes the temporal evolution of Shore D hardness for all investigated materials. HARZ Labs Dental Clear exhibited the greatest aging-related reduction in hardness, whereas Duran PETG remained mechanically stable throughout the experimental period.

### 3.2. Three-Point Bending Test

Flexural modulus, flexural strength, and strain at failure were assessed for all materials and aging conditions. Results are summarized by material in Table 10, Table 11, Table 12 and Table 13.

Material C (Duran—PETG):

Material C exhibited complete fracture behavior under three-point bending in all aging conditions. Flexural modulus showed a transient decrease after 24 h followed by recovery above baseline at 48 and 72 h (Table 10). One-way ANOVA demonstrated significant differences (F = 25.27; *p* < 0.001), with post hoc analysis identifying C48 as significantly different from baseline and C24. Flexural strength followed a similar trend, whereas no statistically significant differences were observed for strain at failure (*p* = 0.174).

**Figure 3:**
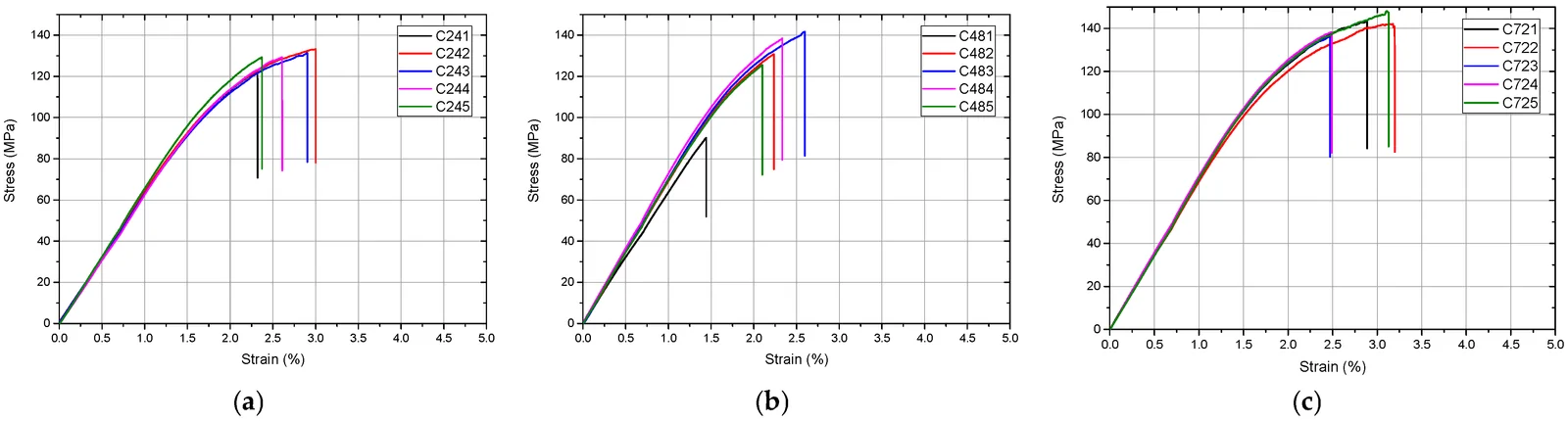
Stress–strain curves obtained from three-point bending tests for Duran (C) after (**a**) 24 h, (**b**) 48 h, and (**c**) 72 h of accelerated aging. Each curve corresponds to an individual specimen (*n* = 5 per condition).

The stress–strain behavior of material C is illustrated in Figure 3.

Material F (Bilkim Polywax—PMMA):

Flexural modulus did not differ significantly among aging groups (*p* = 0.243) (Table 11). Flexural strength exhibited significant differences (*p* = 0.031), with post hoc analysis identifying differences only between F48 and F72. No statistically significant differences were observed for strain at failure.

**Figure 4:**
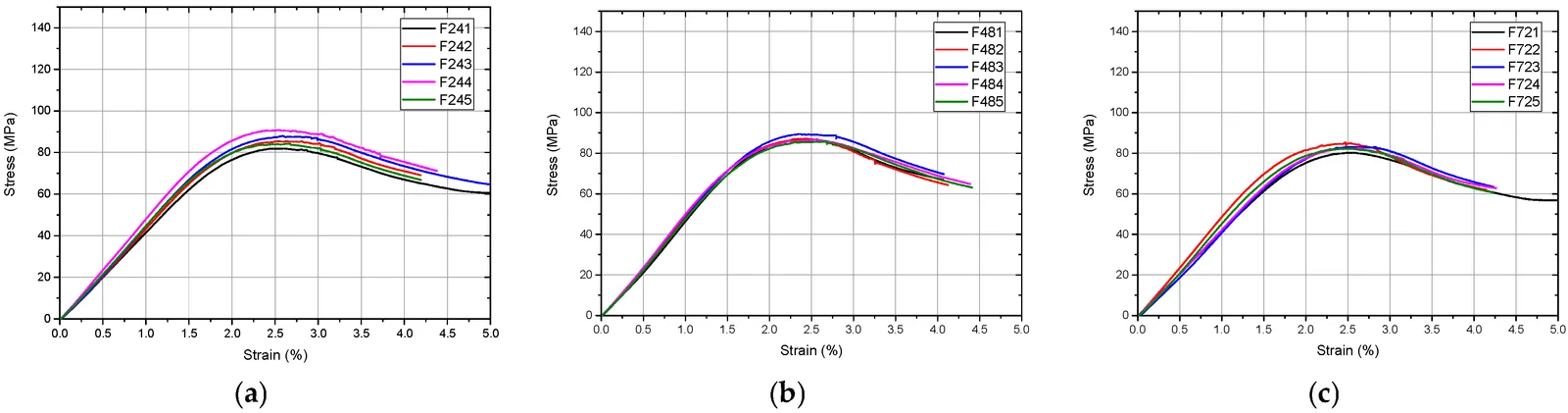
Stress–strain curves obtained from three-point bending tests for Bilkim Polywax (F) after (**a**) 24 h, (**b**) 48 h, and (**c**) 72 h of accelerated aging. Each curve corresponds to an individual specimen (*n* = 5 per condition).

The mechanical response of material F under different aging conditions is presented in Figure 4.

Material H (HARZ Labs Dental Clear—SLA resin):

Material H exhibited the greatest aging-related deterioration among all investigated materials (Table 12). Flexural modulus decreased by approximately 49%, while flexural strength decreased by approximately 71% after 72 h compared with baseline. Significant differences were identified for all evaluated parameters (all *p* < 0.001), although post hoc analysis revealed no significant difference between H24 and H48 for flexural strength.

**Figure 5:**
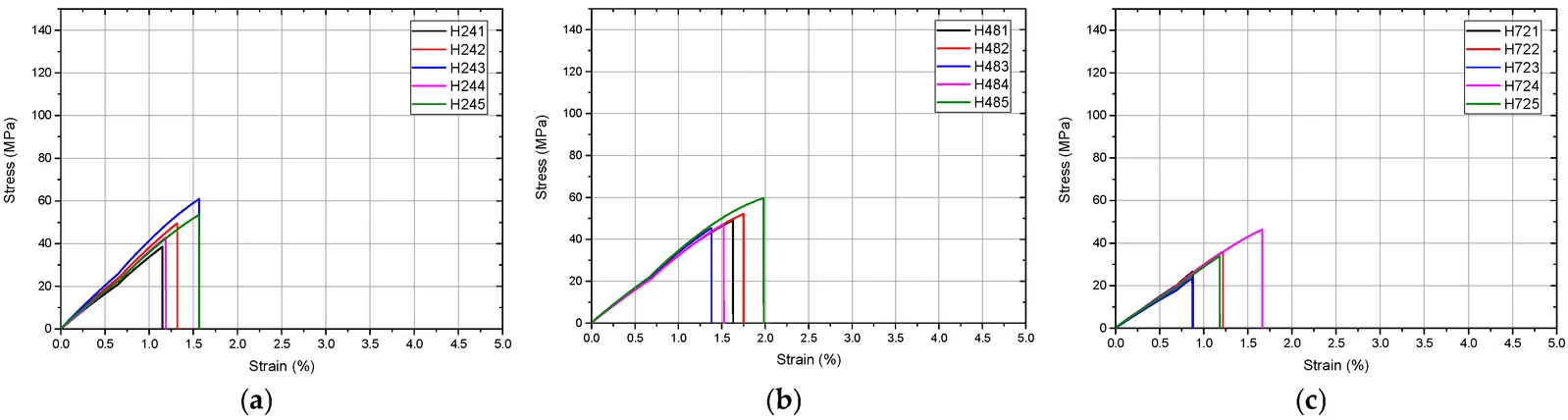
Stress–strain curves obtained from three-point bending tests for HARZ Labs Dental Clear (H) after (**a**) 24 h, (**b**) 48 h, and (**c**) 72 h of accelerated aging. Each curve corresponds to an individual specimen (*n* = 5 per condition).

The stress–strain curves for material H are shown in Figure 5, highlighting the pronounced effect of accelerated aging.

**Figure 6:**
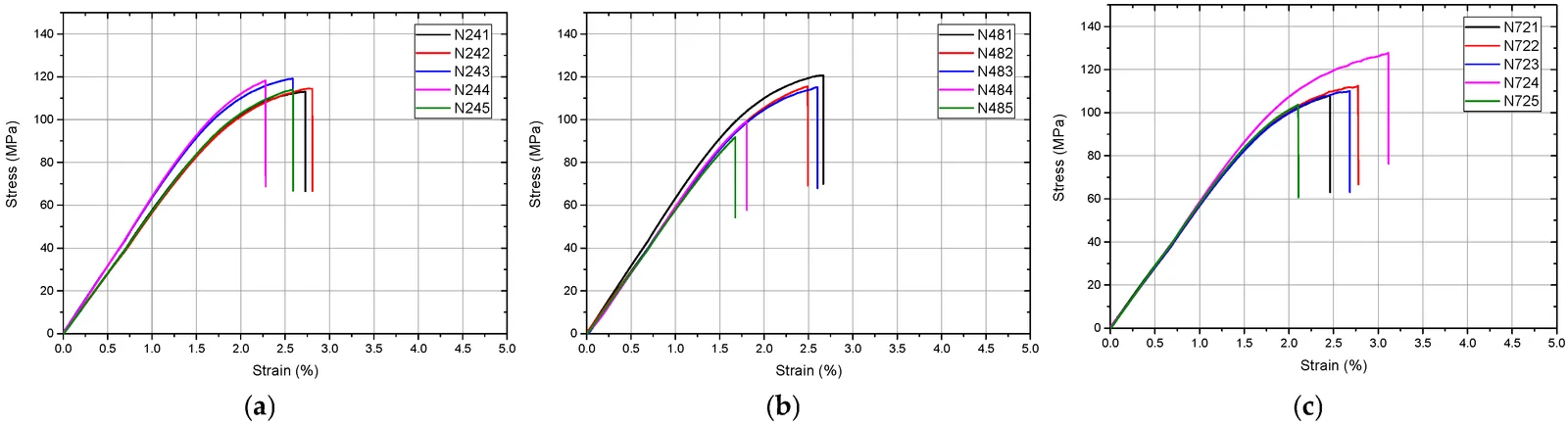
Stress–strain curves obtained from three-point bending tests for NextDent Ortho Rigid (N) after (**a**) 24 h, (**b**) 48 h, and (**c**) 72 h of accelerated aging. Each curve corresponds to an individual specimen (*n* = 5 per condition).

Material N (NextDent Ortho Rigid—SLA resin):

Material N exhibited complete specimen fracture under all aging conditions (Table 13). Significant differences were observed only for flexural modulus (ANOVA, *p* = 0.006), with post hoc analysis identifying a difference between NR and N72 (*p =* 0.005). Flexural strength and strain at failure (Figure 6) were not significantly affected by aging (*p =* 0.411 and *p =* 0.383, respectively).

Table 14 summarizes the relative changes observed after 72 h of accelerated aging compared with baseline values.

## 4. Discussion

The null hypothesis of this study stated that no statistically significant differences in mechanical properties would be observed between baseline and post-aging measurements across the tested materials at different time points. Based on the results obtained, the null hypothesis was partially rejected. Statistically significant changes were identified for all four materials in at least one mechanical parameter, confirming that accelerated aging in artificial saliva at 60 °C exerts material-dependent effects on the mechanical behavior of polymeric splint materials. The nature, magnitude, and time dependence of these changes differed substantially among the tested groups, which constitute the central finding of the present study.

Duran (C), the thermoformed PETG material, exhibited the greatest dimensional stability across all evaluated parameters. Shore D hardness showed no statistically significant changes throughout the aging period (*p* = 0.651), consistent with the known chemical resistance and low water absorption of PETG. The observed variation in flexural modulus, which initially decreased at 24 h and subsequently increased beyond baseline values at 48 and 72 h, warrants interpretation with caution. This non-monotonic pattern may reflect competing hydrothermal effects occurring during early and prolonged exposure, including transient plasticization phenomena and possible thermally induced structural rearrangements within the polymer matrix. However, no physicochemical analyses were performed to directly confirm these mechanisms. The absence of statistically significant changes in strain at failure further supports the overall mechanical stability of this material. These findings align with reports of PETG maintaining dimensional and mechanical integrity under conditions representative of short- to medium-term intraoral use [5,6].

Bilkim Polywax PMMA (F) demonstrated a selective aging response. Shore D hardness showed a statistically significant reduction after 48 h (*p =* 0.004), returning to the 24 h level at 72 h without recovering to baseline, suggesting that superficial mechanical properties were transiently affected by water sorption-mediated plasticization. PMMA is known to absorb water progressively, with absorbed molecules acting as plasticizers that reduce polymer chain mobility and surface hardness [20,21]. The ductile deformation behavior observed throughout all conditions is consistent with the viscoelastic properties of conventionally processed PMMA under three-point loading. Flexural strength showed a statistically significant change between F48 and F72, indicating that extended aging may reduce the structural resistance of the material under bending, potentially as a result of hydrothermal degradation phenomena within the polymer matrix [22].

Previous investigations on CAD/CAM PMMA materials have similarly suggested that polymerization quality and microstructural characteristics influence resistance to aqueous degradation and mechanical softening [23,24,25,26].

The absence of significant changes in flexural modulus across aging conditions suggests that elastic stiffness is less sensitive to short-term aqueous exposure than peak load-bearing capacity.

HARZ Labs Dental Clear (H) demonstrated the most pronounced and consistent mechanical degradation among the tested materials. Progressive reductions in Shore D hardness, flexural modulus, flexural strength, and strain at failure were observed across all aging intervals, with statistically significant differences confirmed for all parameters (*p <* 0.001). The reduction in flexural modulus exceeded 49% by 72 h relative to baseline, and flexural strength was reduced by more than 70% at the same time point, corresponding to a residual value of approximately 29% of the initial baseline. This magnitude of deterioration indicates a marked reduction in mechanical performance under the accelerated aging conditions investigated in the present study. The progressive and continuous nature of the degradation, as confirmed by post hoc analyses showing significant differences between all-time points for most parameters, indicates an ongoing structural process rather than a threshold-limited plasticization effect [27,28,29].

Possible explanations for the observed degradation may include hydrothermal softening, water-mediated disruption of the polymer network, and alterations in the integrity of the cross-linked resin structure. However, these mechanisms were not directly evaluated in the present study and should therefore be interpreted cautiously [30,31,32]. The fractographic observations, which revealed a transition from surfaces decorated with microcracks and river markings to smoother fracture planes with increasing aging, are consistent with a progressive reduction in energy dissipation capacity and an increasingly brittle failure mode.

NextDent Ortho Rigid (N) displayed an intermediate aging response. The initial drop in Shore D hardness at 24 h (approximately 1.5%), followed by stabilization of the aged subgroups, suggests a rapid early-phase response to aqueous exposure, potentially associated with superficial hydrothermal interactions within the resin matrix, followed by subsequent stabilization. This pattern differs from the progressive degradation observed for material H and may reflect the specific cross-link density and monomer composition of this resin, which is formulated and certified for intraoral use as a dental appliance material [9,33]. The statistically significant reduction in flexural modulus between baseline and the 72 h subgroup (*p* = 0.005–0.006), in the absence of significant changes in flexural strength and strain at failure, suggests that elastic stiffness may be more sensitive to aging in this material than peak structural resistance. This observation is clinically relevant, as a reduction in flexural modulus implies increased deformability under functional loading, which may affect the precision of fit and occlusal contact distribution of fabricated splints [12,13,34].

The structural characteristics of SLA-printed splint resins may further contribute to their aging behavior at the microstructural level; Differences in internal microstructural organization between printed and milled polymers may also contribute to variations in aging behavior reported in the literature [35].

The comparative analysis across materials highlights a clear hierarchy of aging resistance under the conditions tested. Duran (C) demonstrated the greatest stability, followed by NextDent Ortho Rigid (N) and Bilkim Polywax PMMA (F), while HARZ Labs Dental Clear (H) exhibited the most pronounced degradation. This ordering does not correspond directly to manufacturer-reported baseline mechanical properties, which underscores the importance of evaluating aging behavior independently from initial performance characteristics [29,36]. In particular, the high initial values of Shore D hardness and flexural strength reported for material H did not translate into resistance to accelerated aging, suggesting that photopolymer architecture and cross-link stability under hydrothermal conditions may be the primary determinants of aging resistance in SLA-printed materials [30,32,37].

Previous studies using FT-IR and nanoindentation analyses have suggested that polymerization efficiency and cross-link density may influence the long-term mechanical stability of methacrylate-based dental materials [38]. Although such physicochemical parameters were not evaluated in the present study, they may partially explain the different aging responses observed among the investigated SLA resins.

The present study introduces several methodological contributions. It provides a comparative assessment of four materials spanning the principal fabrication categories currently in clinical use, within a single standardized aging framework. The use of multiple post hoc correction methods increases the robustness of statistical inference and reduces the risk of type I errors in multiple comparison contexts. The evaluation of three distinct mechanical parameters in addition to hardness enables a more complete characterization of aging-related behavioral changes, as different parameters may exhibit different sensitivities to the same degradation process.

A limitation of this study was the use of standardized rectangular specimens rather than clinically representative splint geometries, which may limit the direct extrapolation of findings to full-arch appliances. The Shore D measurements were obtained using an analog durometer with a mechanical resolution of 0.5 units; although the use of ten determinations per condition and averaging mitigates this constraint, future studies should consider digital or electronic durometers for improved precision, particularly when evaluating materials with narrow inter-group differences. A limitation of the present study is that the aging protocol does not allow direct extrapolation to a defined period of intraoral service. The elevated-temperature immersion model provides a standardized comparative hydrothermal challenge, but it cannot fully reproduce the complex clinical environment, including cyclic loading, thermal fluctuations, biofilm activity, salivary enzymes, dietary acids, and patient-specific wear patterns. The sample size of five specimens per subgroup for bending evaluation, while consistent with similar in vitro investigations, may limit statistical power for the detection of small effect sizes. Additionally, no surface roughness or color stability assessments were performed, which would provide complementary information regarding material deterioration.

Additionally, no physicochemical characterization methods such as water sorption analysis, residual monomer quantification, FTIR spectroscopy, DSC, SEM, or microstructural evaluation were performed. Consequently, the mechanistic interpretations proposed for the observed aging-related mechanical changes remain hypothetical and literature-based.

Within the limitations of this in vitro study, accelerated aging produced material-dependent changes in the investigated mechanical properties. The findings suggest that ageing resistance may represent an important consideration when comparing materials intended for occlusal splint fabrication. However, further investigations incorporating clinically relevant splint geometries, cyclic loading, thermocycling, pH variation, and long-term clinical evaluation are required before definitive clinical recommendations can be made.

## 5. Conclusions

Within the limitations of this in vitro study, the following conclusions can be drawn:

1. Duran (PETG thermoformed material) demonstrated the greatest mechanical stability under accelerated aging conditions, with no statistically significant changes in Shore D hardness across all evaluated time points and non-progressive flexural behavior. These findings suggest favorable aging resistance compared with the other investigated materials under the present experimental conditions
2. Bilkim Polywax PMMA exhibited selective aging-related changes, including a transient reduction in Shore D hardness at 48 h and a statistically significant decrease in flexural strength at 72 h. The ductile deformation behavior and absence of catastrophic failure suggest a comparatively stable mechanical response under bending despite measurable aging-related alterations.
3. HARZ Labs Dental Clear (SLA photopolymer resin) demonstrated the most pronounced and progressive mechanical deterioration among all investigated materials, with statistically significant reductions in Shore D hardness, flexural modulus, flexural strength, and strain at failure across all aging intervals. Under the present experimental conditions, reductions in flexural modulus exceeding 49% and in flexural strength exceeding 70% after 72 h indicated substantial susceptibility to hydrothermal aging.
4. NextDent Ortho Rigid (SLA photopolymer resin formulated for dental appliance use) exhibited an initial hardness drop at 24 h followed by stabilization, and a statistically significant reduction in flexural modulus at 72 h without corresponding changes in flexural strength or strain at failure. Compared with HARZ Labs Dental Clear, this material demonstrated a more stable aging profile under the investigated conditions.
5. Comparative analysis across materials demonstrates that the two investigated SLA-printed photopolymer resins showed greater susceptibility to accelerated hydrothermal aging than the thermoformed PETG or milled PMMA materials evaluated in this study, although the magnitude and progression of degradation differed substantially between the two printed resins. The findings emphasize the importance of assessing aging resistance independently from baseline mechanical properties when comparing materials intended for digitally fabricated dental appliances.
6. The present findings were obtained using standardized rectangular specimens under controlled laboratory conditions and therefore should not be interpreted as direct predictors of long-term clinical performance of a full-arch occlusal splint.
7. Future research should investigate the cumulative effects of repeated thermal cycling combined with mechanical fatigue loading on the properties of these materials, incorporate full 3D surface deviation analysis and surface roughness evaluation after aging, and extend the assessment to a broader range of commercially available SLA resins and milled polymers. Long-term clinical studies correlating in vitro aging performance with intraoral material behavior and patient outcomes would further strengthen the evidence base for material selection in digitally fabricated occlusal splint therapy.

## Figures and Tables

**Table 1:** Material composition and manufacturer-specified properties.

Material (Code)	Type	Composition	Manufacturing Method	Flexural Strength (MPa)	Flexural Modulus (MPa)	Other Properties
Duran (C)	Thermoplastic	PETG (polyethylene terephthalate glycol)	Thermoforming	-	-	Low water absorption; high fracture resistance; biocompatible
Bilkim Polywax (F)	PMMA	Polymethylmethacrylate (PMMA)	CAD/CAM milling	~105.91	-	Water absorption: 13.40 µg/mm^3^; solubility: 0.87 µg/mm^3^
HARZ Labs Dental Clear (H)	Photopolymer resin	Methacrylate-based photopolymer	SLA 3D printing	120.0 ± 20.0	2000 ± 156	Shore D: 85 ± 3; low shrinkage (<0.5%)
NextDent Ortho Rigid (N)	Photopolymer resin	Methacrylate-based photopolymer	SLA 3D printing	≥78	2075	Water absorption: 20 µg/mm^3^; solubility: 0.8 µg/mm^3^; Class IIa biocompatible

**Table 2:** Descriptive statistics for Shore D hardness—Material C (Duran).

	Shore D			
	CR	C24	C48	C72
Valid	10	10	10	10
Median	97.00	97.00	97.00	97.00
Mean	97.10	96.90	97.25	96.80
SD	0.568	0.568	0.540	1.418
Min	96.00	96.00	96.50	93.00
Max	98.00	98.00	98.00	98.00

**Table 3:** One-way ANOVA results for Shore D hardness—Material C.

Cases	Sum of Squares	df	Mean Square	F	*p*
Sample	1.219	3	0.406	0.551	0.651
Residuals	26.53	36	0.737		

**Table 4:** Descriptive statistics for Shore D hardness—Material F (Bilkim Polywax PMMA).

	Shore D			
	FR	F24	F48	F72
Valid	10	10	10	10
Median	89.00	88.00	87.50	88.50
Mean	89.25	88.20	87.40	88.20
SD	0.635	0.919	1.265	1.229
Min	88.00	87.00	85.00	86.00
Max	90.00	90.00	89.00	90.00

**Table 5:** One-way ANOVA results for Shore D hardness—Material F.

Cases	Sum of Squares	df	Mean Square	F	*p*
Sample	17.27	3	5.756	5.283	0.004
Residuals	39.22	36	1.090		

**Table 6:** Descriptive statistics for Shore D hardness—Material H (HARZ Labs Dental Clear).

	Shore D			
	HR	H24	H48	H72
Valid	10	10	10	10
Median	95.00	92.00	90.75	89.50
Mean	95.10	92.20	90.70	89.60
SD	0.516	0.587	0.919	0.966
Min	94.00	91.00	89.00	88.00
Max	96.00	93.00	92.00	91.00

**Table 7:** One-way ANOVA results for Shore D hardness—Material H.

Cases	Sum of Squares	df	Mean Square	F	*p*
Sample	170.6	3	56.87	95.22	<0.001
Residuals	21.50	36	0.597		

**Table 8:** Descriptive statistics for Shore D hardness—Material N (NextDent Ortho Rigid).

	Shore D			
	NR	N24	N48	N72
Valid	10	10	10	10
Median	96.00	94.75	95.00	95.00
Mean	96.05	94.65	94.85	94.85
SD	0.369	0.530	0.818	0.530
Min	95.50	94.00	94.00	94.00
Max	97.00	95.50	96.00	95.50

**Table 9:** One-way ANOVA results for Shore D hardness—Material N.

Cases	Sum of Squares	df	Mean Square	F	*p*
Sample	12.30	3	4.100	12.00	<0.001
Residuals	12.30	36	0.342		

**Table 10:** Descriptive statistics for flexural properties—Material C (Duran).

	Flexural Modulus [MPa]				Flexural Strength [MPa]				Strain at Failure [%]			
	CR	C24	C48	C72	CR	C24	C48	C72	CR	C24	C48	C72
*n*	5	5	5	5	5	5	5	5	5	5	5	5
Mean	3295	3174	3546	3495	133.1	129.1	134.1	141.6	2.576	2.640	2.318	2.836
SD	100.6	87.46	52.57	57.83	8.689	3.979	6.324	4.584	0.492	0.307	0.183	0.345
Min	3162	3085	3476	3423	123.3	122.6	125.6	136.3	2.017	2.316	2.100	2.470
Max	3402	3303	3622	3563	142.7	133.2	141.7	148.0	3.080	3.000	2.600	3.200

**Table 11:** Descriptive statistics for flexural properties—Material F (Bilkim Polywax PMMA).

	Flexural Modulus [MPa]				Flexural Strength [MPa]				Strain at Failure [%]			
	FR	F24	F48	F72	FR	F24	F48	F72	FR	F24	F48	F72
*n*	5	5	5	5	5	5	5	5	5	5	5	5
Mean	2249	2271	2389	2200	84.26	86.08	87.28	82.70	4.430	4.596	4.212	4.372
SD	165.5	123.8	119.7	163.9	2.132	3.409	1.335	1.808	0.391	0.481	0.174	0.342
Min	2030	2139	2213	2008	81.29	81.94	85.84	80.26	4.170	4.190	4.060	4.150
Max	2402	2466	2503	2450	86.30	90.78	89.48	85.30	5.120	5.210	4.410	4.980

**Table 12:** Descriptive statistics for flexural properties—Material H (HARZ Labs Dental Clear).

	Flexural Modulus [MPa]				Flexural Strength [MPa]				Strain at Failure [%]			
	HR	H24	H48	H72	HR	H24	H48	H72	HR	H24	H48	H72
*n*	5	5	5	5	5	5	5	5	5	5	5	5
Mean	2769	1801	1586	1405	114.5	49.04	50.68	33.29	2.522	1.574	1.744	1.248
SD	127.5	147.8	49.89	83.24	4.180	8.793	5.594	8.899	0.366	0.265	0.235	0.329
Min	2570	1640	1539	1301	110.5	38.52	45.38	23.55	2.170	1.350	1.450	0.930
Max	2916	2017	1651	1523	119.3	60.90	59.64	46.34	3.070	1.980	2.070	1.730

**Table 13:** Descriptive statistics for flexural properties—Material N (NextDent Ortho Rigid).

	Flexural Modulus [MPa]				Flexural Strength [MPa]				Strain at Failure [%]			
	NR	N24	N48	N72	NR	N24	N48	N72	NR	N24	N48	N72
*n*	5	5	5	5	5	5	5	5	5	5	5	5
Mean	3149	2968	3007	2862	117.4	115.8	108.5	112.4	2.528	2.600	2.250	2.630
SD	110.6	154.9	109.1	52.68	7.837	2.710	12.29	9.175	0.390	0.202	0.468	0.376
Min	2958	2835	2885	2790	104.0	113.1	91.84	103.7	1.960	2.280	1.680	2.110
Max	3229	3150	3160	2926	123.1	119.2	120.7	127.8	2.970	2.810	2.670	3.120

**Table 14:** Relative change after 72 h of accelerated aging compared with baseline values.

Material	Shore D Hardness Δ%	*p*-Value	Flexural Modulus Δ%	*p*-Value	Flexural Strength Δ%	*p*-Value	Strain at Failure Δ%	*p*-Value
Duran (C)	−0.3	0.651	+6.1	-	+6.4	-	+10.1	-
Bilkim PMMA (F)	−1.2	0.004	−1.3	0.243	−1.9	0.031	−2.2	-
HARZ Dental Clear (H)	−5.8	<0.001	−49.3	<0.001	−70.9	<0.001	−50.5	<0.001
NextDent Ortho Rigid (N)	−1.3	<0.001	−9.1	0.008	−4.3	0.411	+4.0	0.383

## Data Availability

The data presented in this study are available from the corresponding author upon reasonable request. The data are not publicly available because they form part of ongoing research.
